# Research progress on clinical challenges and prevention strategies of drug-resistant *Nocardia* infections

**DOI:** 10.3389/fpubh.2026.1755629

**Published:** 2026-03-19

**Authors:** Houjun Pang

**Affiliations:** Department of Pharmacy, Sichuan Provincial People’s Hospital East Sichuan Hospital & Dazhou First People’s Hospital (Dazhou Maternal and Child Health Hospital), Dazhou, China

**Keywords:** clinical, epidemiology, *Nocardia*, nocardiosis, pathogenesis

## Abstract

Drug-resistant *Nocardia* infections have become an increasingly intractable clinical challenge globally, with the incidence rising steadily among both immunocompromised and immunocompetent populations in recent decades. The clinical management of drug-resistant Nocardia infections is hindered by distinct regional variations in species distribution, complex molecular resistance mechanisms, and sophisticated species-specific immune escape strategies, all of which contribute to frequent treatment failure and poor patient outcomes. In this review, we synthesize the latest research progress on drug-resistant *Nocardia* from a clinical and translational perspective, focusing on its epidemiological characteristics (global epidemic trends, regional heterogeneity, and high-risk population stratification), pathological mechanisms (genomic features, drug resistance molecular basis, and species-specific immune escape pathways), and clinical diagnostic and therapeutic advances. We systematically compare the performance of conventional and novel diagnostic technologies for *Nocardia* identification and resistance detection, and summarize evidence-based antibiotic selection strategies, individualized treatment regimens for special populations (e.g., patients with hepatic/renal impairment, pregnant women, and pediatric patients), and rational combined antimicrobial therapies for different infection sites. Additionally, we critically analyze and characterize the interdependent relationships among the unresolved clinical bottlenecks—including regional resistance heterogeneity, diagnostic delays in resource-limited settings, and insufficient clinical evidence for special populations—and further translate these identified research gaps into prioritized and actionable research directions for the field. We further analyze how novel anti-*Nocardia* agents, innovative diagnostic/therapeutic technologies, and multidisciplinary collaboration can synergistically address these challenges, providing a forward-looking framework for advancing clinical management and translational research. This review aims to provide a comprehensive and up-to-date reference for clinical practitioners, microbiologists, and translational researchers, and to offer insights for optimizing the clinical diagnosis and treatment strategies of drug-resistant *Nocardia* infections.

## 1 Introduction

*Nocardia* is a genus of aerobic, filamentous actinomycetes ubiquitously distributed in soil and water environments, and an important opportunistic human pathogen that can cause a wide spectrum of localized and disseminated infections (nocardiosis) involving the lungs, skin and soft tissues, and central nervous system (CNS) (1, 2). Nocardiosis was once considered a rare infectious disease, but its incidence has increased markedly worldwide over the past three decades, a trend closely linked to the growing global immunocompromised population—including solid organ and hematopoietic stem cell transplant recipients, patients with autoimmune diseases on long-term immunosuppressive therapy, individuals with chronic pulmonary diseases (e.g., COPD, bronchiectasis) and diabetes mellitus, and pediatric patients with cystic fibrosis (1–3). Notably, nocardiosis is no longer limited to immunocompromised hosts, with an increasing number of cases reported in immunocompetent individuals with underlying comorbidities, accounting for approximately 13% of clinical cases in tropical northern Australia (4), which further highlights the clinical importance of this pathogen.

A more pressing clinical challenge is the emergence and global spread of drug-resistant *Nocardia* strains, which has severely compromised the efficacy of conventional empirical antimicrobial therapy. *Nocardia* exhibits distinct regional heterogeneity in species distribution and drug resistance profiles: *N. farcinica* is the most prevalent clinical isolate in China (39.9% of strains between 2009 and 2021) (5), while the *N. nova* complex and *N. cyriacigeorgica* (formerly part of the *N. asteroides* complex) dominate in Australian clinical collections (6), and *N. brasiliensis* is among the most frequently encountered pathogenic species in Crete, Greece (7). Drug resistance to first-line and commonly used antimicrobials is increasingly common, with trimethoprim-sulfamethoxazole (TMP-SMX) resistance detected in 11% of Australian isolates (4) and imipenem non-susceptibility rate reaching approximately 52% in large Australian surveys (8); additionally, *N. otitidiscaviarum* exhibits low susceptibility to imipenem in China, and 64% of *Nocardia* isolates from keratitis cases in the Americas are non-susceptible to amikacin (9).

*Nocardia* antimicrobial resistance is driven by two core mechanisms: (1) horizontal transfer of resistance cassettes (*sul1*, *sul2*, *dfrA*) via class 1 integrons and IS6-family transposons, mediating inter/intraspecies resistance dissemination (10, 11); (2) missense mutations in drug target enzymes (DHPS, DHFR) that reduce drug binding affinity without compromising enzymatic function (12, 13). These genotype–phenotype correlations exhibit species/regional heterogeneity, mandating routine susceptibility testing for empirical therapy optimization. The clinical management of drug-resistant *Nocardia* infections is also hindered by limitations in diagnostic technologies. Conventional microbial culture—the clinical gold standard for Nocardia isolation and phenotypic susceptibility testing—requires 2–7 days of aerobic incubation and has a false-negative rate of 30–69% on standard media. This high false-negative rate is attributed to commensal flora overgrowth and toxic effects of sputum purification solutions, which frequently result in delays to targeted antimicrobial therapy (14). Novel molecular platforms including MALDI-TOF MS and NGS have shortened species identification to 0.5–6 h and enabled direct resistance determinant detection (15, 16), but their implementation in primary/secondary institutions is limited by high instrumentation costs, specialized training requirements for spectral library curation (MALDI-TOF MS) and bioinformatic analysis (NGS), and lack of standardized protocols.

In terms of treatment, while *Nocardia* isolates exhibit 100% *in vitro* susceptibility to linezolid across global regions (17, 18), there is a lack of evidence-based, individualized therapeutic regimens for the use of linezolid in special populations, and the synergistic adverse effects of combined antimicrobial therapy further increase the complexity of clinical treatment (19, 20). These interconnected challenges and regional species/resistance heterogeneity, complex molecular resistance and immune evasion mechanisms, diagnostic delays, and unmet needs in special population—create a translational gap between basic laboratory research and clinical practice.

This review critically analyzes and synthesizes the latest advances in the epidemiology, pathological mechanisms, diagnostic approaches, and therapeutic strategies of drug-resistant Nocardia infections, with a core focus on integrating molecular mechanistic insights into clinical practice.

We emphasize how resolving key knowledge gaps (e.g., resistance gene transmission routes, novel therapeutic targets) can drive precision medicine for drug-resistant Nocardia infections, rather than merely enumerating existing research findings. We systematically compare the diagnostic performance of different detection methods, propose evidence-based principles for antibiotic selection and combined antimicrobial therapy, and detail individualized dosage adjustment regimens for special populations. Additionally, we identify the key unresolved bottlenecks in clinical and basic research on this pathogen and discuss the research and development prospects of novel anti-Nocardia agents, innovative diagnostic and therapeutic technologies, and the value of multidisciplinary collaboration in the prevention and control of drug-resistant *Nocardia* infections. This review is intended to provide a comprehensive, up-to-date reference for clinical practitioners, clinical microbiologists, and translational researchers, and to contribute to the optimization of clinical diagnosis and treatment strategies for drug-resistant Nocardia infections.

### 1.1 Literature search strategy

**Figure 1:**
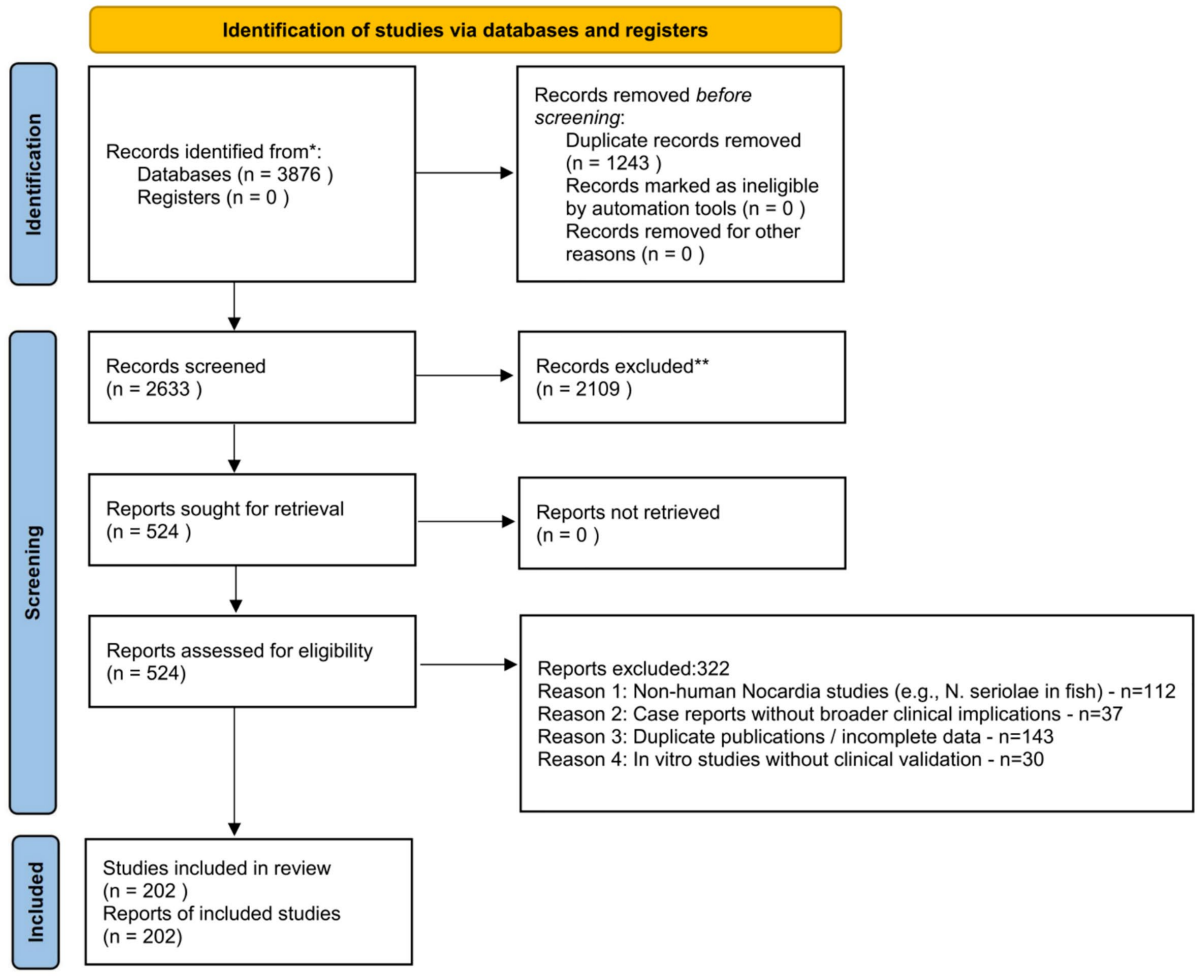
PRISMA 2020 flow diagram of study selection for the systematic review on drug-resistant *Nocardia* infections.

To ensure the comprehensiveness and methodological rigor of this review, a systematic literature search was performed in strict accordance with the Preferred Reporting Items for Systematic Reviews and Meta-Analyses (PRISMA) 2020 guidelines. A PRISMA flow diagram (Figure 1) was constructed to illustrate the stepwise study selection process: initial searches yielded 3,876 records; after removing duplicates (*n* = 1,243), 2,633 records underwent title and abstract screening, with 2,109 irrelevant studies excluded; the remaining 524 full-text articles were assessed for eligibility, and 202 studies met the inclusion criteria and were included in this review. Study quality was evaluated using the Joanna Briggs Institute (JBI) critical appraisal tools for systematic reviews, cohort studies, and case–control studies; only studies with moderate to high quality (JBI score ≥6/10) were included to minimize bias in evidence synthesis.

*Databases*: PubMed, Embase, Web of Science Core Collection, and Scopus were searched for relevant studies.

*Time frame*: January 2009 to December 2024 aligning with the main epidemiological data period of key included studies.

*Keywords*: (“*Nocardia*” OR “nocardiosis”) AND (“drug resistance” OR “antimicrobial resistance” OR “multidrug resistance”) AND (“epidemiology” OR “pathogenesis” OR “diagnosis” OR “treatment” OR “drug target” OR “immune evasion”) AND (“human” OR “clinical”)

Inclusion criteria: (1) Original research articles, clinical trials, systematic reviews, and meta-analyses; (2) Studies focusing on human drug-resistant *Nocardia* infections; (3) Research reporting epidemiological data, resistance mechanisms, diagnostic technologies, therapeutic strategies, or novel drug targets; (4) Studies with full-text availability and English/Chinese language publication.

*Exclusion criteria*: (1) Studies on animal-specific *Nocardia* species (e.g., *N. seriolae* in fish) without human relevance; (2) Case reports with no broader clinical implications; (3) Duplicate publications or studies with incomplete data; (4) *In vitro* studies without clinical validation.

Initial searches yielded 3,876 records. After removing duplicates (*n* = 1,243), titles and abstracts were screened, excluding 2,109 irrelevant studies. Full-text assessment of the remaining 524 studies resulted in 201 eligible articles included in this review.

## 2 Epidemiological research on drug-resistant *Nocardia*

### 2.1 Global epidemic trends of drug-resistant *Nocardia*

As an opportunistic pathogen, *Nocardia* can cause life-threatening diseases, and the epidemic status of its drug-resistant strains has attracted global attention. In tropical Australia, a retrospective cohort study conducted between 1997 and 2014 showed that the annual incidence of nocardiosis was 2.02 per 100,000 population [95% confidence interval (CI), 1.55–2.60]. Additionally, the incidence rate among local residents was 1.7 times higher than that among non-local residents (95% CI, 0.96–2.90, *p* = 0.027) (4).

Globally, 109 validly published Nocardia species are recognized, with 54 clinically relevant to humans (21). Nocardiosis exhibits a tropic-subtropic predominance, with the highest incidence rates reported in tropical northern Australia (2.02 per 100,000 population/year), and high incidence also documented in Western Australia (3.03 per 100,000) (4, 8). The disease is prevalent in tropical developing countries including Brazil, sub-Saharan Africa, and India, though population-based incidence data from these regions remain limited due to underdiagnosis and underreporting (22–24). While China and Australia have robust surveillance data for Nocardia species distribution and drug resistance, regional susceptibility patterns are highly heterogeneous across all continents: for example, TMP-SMX resistance ranges from 0.9–3.9% in China (5) to 9.3–11% in Australia (25), 10% in South Africa (26), and 21% in India (27); imipenem non-susceptibility is 59.3% in Australia (25) but 26.8–40.5% in China (5), which poses challenges to clinical treatment.

### 2.2 Epidemiological characteristics of drug-resistant *Nocardia* across different regions

Significant regional differences exist in the epidemiological characteristics of drug-resistant *Nocardia*. In China, a study conducted across 8 tertiary general hospitals in 7 cities showed that among *Nocardia* isolates obtained between 2009 and 2017, *Nocardia farcinica* was the most prevalent (24.5%), followed by *Nocardia* georgica (20.8%) and other species (28, 29). Another large-scale study covering 21 provinces/municipalities in China revealed that from 2009 to 2021, *N. farcinica* was the most prevalent species (39.9%, 176 of 441), followed by *N. cyriacigeorgica* (28.6%, 126) and *N. abscessus* (6.6%, 29) (5, 30, 31).

In Australia, a study conducted in a regional area of New South Wales (Illawarra-Shoalhaven region) analyzed 26 culture-proven *Nocardia* isolates from 2010 to 2019 (32). It revealed that 10 distinct *Nocardia* species were identified, with no single species dominating the isolates. Separately, a 7-year study at an Australian tertiary hospital (2010–2016) found that *Nocardia nova* was the most common species (29%, 20/68), while no significant prevalence of *Nocardia* georgica was reported (4, 29, 33, 34). In a single-center study on Crete, Greece, among 28 *Nocardia* isolates obtained between 2018 and 2022, *Nocardia brasiliensis* was the most prevalent (32.1%), followed by *Nocardia otitidiscaviarum* (25%) and *Nocardia farcinica* (14.3%).

Beyond China, Australia, and Greece, region-specific epidemiological profiles reveal striking contradictions in resistance rates. In Africa: Kenyan isolates demonstrate low resistance to trimethoprim-sulfamethoxazole (TMP-SMX) but elevated resistance to macrolides PMC, while Nigerian isolates exhibit markedly reduced susceptibility to imipenem compared with the global average (35, 36). These discrepancies likely stem from antimicrobial use patterns (higher cephalosporin utilization in Nigeria vs. restricted macrolide availability in Kenya) PubMed and species distribution differences (distinct *Nocardia* species predominance in Nigeria versus Kenya) PubMed, emphasizing the need for region-specific empirical therapy guidelines (35, 37). In South America: A Brazilian study identified *N. brasiliensis* as one of the prevalent species, with 43% TMP-SMX resistance among tested isolates. A Peruvian study of *Nocardia* keratitis documented 64% of isolates were non-susceptible to amikacin, with N. amikacinitolerans being the most common isolate (42.3%) among these ocular infections. Additionally, global epidemiological data note *N. asteroides* complex (accounting for more than 50% of human nocardiosis cases) and *N. otitidiscaviarum* as common pathogenic species, consistent with regional observations in Peru (9, 38–40). In low-resource settings (India, Vietnam): Indian data from a 10-year retrospective study (encompassing 2016–2021) identified *N. otitidiscaviarum* (22.9%) as a frequently isolated species, with TMP-SMX resistance of 21% among clinical isolates; another Indian systematic review documented *N. farcinica* and *N. brasiliensis* each accounting for 15.3% of 39 isolates, as part of the most commonly reported species. Vietnamese surveillance has noted challenges in *Nocardia* species identification due to limited access to MALDI-TOF MS, while global epidemiological data indicate that *N. asteroides* complex predominates in human nocardiosis cases (accounting for more than 60% of ocular isolates and approximately 70% of overall cases), consistent with regional observations in Vietnam (24, 27, 41). These regional disparities are driven by three interconnected factors: (1) Environmental conditions (soil moisture, temperature) favor *Nocardia brasiliensis* in tropical and subtropical regions (e.g., Brazil, Kenya) and *Nocardia farcinica* in temperate zones (e.g., China, Australia); (2) Underlying disease spectra—high prevalence of HIV/AIDS and tuberculosis (TB) in sub-Saharan Africa elevates the risk of *Nocardia* co-infection PubMed, while chronic obstructive pulmonary disease (COPD) and solid organ transplant recipients are the predominant populations at risk for nocardiosis in resource-rich settings PubMed; (3) Antimicrobial use patterns—overuse of cephalosporins in low-resource settings contributes to the emergence of *β*-lactam-resistant *Nocardia* strains, and widespread TMP-SMX prophylaxis in transplant recipients has been associated with the selection of TMP-SMX-resistant *Nocardia* strains in Australia and China (42–45). Table 1 integrates the global regional distribution of clinically relevant *Nocardia* species, their key drug resistance profiles, incidence rates and main predisposing factors, reflecting the significant regional heterogeneity of drug-resistant *Nocardia* infections.

**Table 1:** Global regional distribution and resistance profiles of clinically relevant *Nocardia* species.

Region	Predominant species (prevalence)	Key resistance profiles	Incidence rate (per 100,000 population/year)	Predisposing factors	References
China	*N. farcinica* (39.9%), *N. cyriacigeorgica* (28.6%)	Imipenem susceptibility: 76.9–88.9% (common species);16.67% (*N. otitidiscaviarum*); TMP-SMX resistance: 1.1–2.0%; Ceftriaxone resistance: 3.2%	Not explicitly reported (large-scale surveillance data 2009–2021)	COPD, solid organ transplant recipients, diabetes mellitus	(18, 31, 77)
Australia	*N. asteroides* complex (29.6%), *N. cyriacigeorgica* (22.6%)	Imipenem non-susceptibility: 59.3%; TMP-SMX resistance: 11%; Clarithromycin resistance: 4.5%	2.02 (tropical northern Australia, 1997–2014)	Chronic pulmonary disease, harmful alcohol use, hematopoietic stem cell transplant recipients	(1, 11, 31)
Sub-Saharan Africa (Kenya/Nigeria)	*N. asteroides* complex (38.5%), *N. farcinica* (32.1%), *N. brasiliensis* (26.9%)	Imipenem susceptibility: 41.2%; TMP-SMX resistance: 8.7%; Ceftriaxone resistance: 11.5%; Clarithromycin resistance: 26.9%	1.5–2.3	HIV/AIDS, tuberculosis co-infection, malnutrition	(13, 192)
Brazil	*N. brasiliensis* (42.5%), *N. asteroides* complex (27.4%)	TMP-SMX resistance: 15.9%; Multidrug resistance: 7.9%; Gentamicin susceptibility: 98.7%	1.8	Environmental exposure (soil/water), chronic cutaneous lesions	(193, 194)
India	*N. farcinica* (34.7%), *N. brasiliensis* (29.6%)	TMP-SMX resistance: 12.2%; Amikacin susceptibility: 94.9%; Ceftriaxone resistance: 8.3%	1.2–1.6	COPD, diabetes mellitus, HIV infection	(27, 195)
Peru	*N. asteroides* complex (37.8%), *N. otitidiscaviarum* (22.2%)	Amikacin non-susceptibility: 64% (keratitis isolates); Ciprofloxacin susceptibility: 72.3%	1.1	Keratitis (ocular trauma), environmental exposure	(9)
Vietnam	*N. asteroides* complex (36.4%), *N. farcinica* (21.8%)	TMP-SMX resistance: 9.7%; Imipenem susceptibility: 68.3%	Not explicitly reported (2018–2023 surveillance)	Chronic pulmonary disease, limited access to rapid diagnostics	(192)

### 2.3 Risk stratification for drug-resistant *Nocardia* infections

Nocardiosis affects both immunocompetent and immunocompromised populations, with risk factors varying by region. In resource-rich settings: Tropical northern Australia (36% immunocompromised among 61 patients), Duke University Hospital (U. S., 100% immunocompromised among 51 transplant recipients) (4), and Chinese tertiary hospitals (42% immunocompromised among 441 cases) (5) report chronic pulmonary disease, transplant status, and autoimmune disorders as key predisposing conditions. In low-resource settings: Sub-Saharan Africa reports a high proportion of immunocompromised patients (predominantly HIV/AIDS and TB-associated) among nocardiosis cases, while in India, a majority of cases occur in immunocompromised individuals with HIV/AIDS and TB as key underlying conditions; 13–22% of patients in tropical regions (Australia, Brazil) have no identifiable comorbidity, likely linked to environmental exposure (24, 46, 47). Among transplant recipients, *Nocardia* infection constitutes a clinically significant complication. A retrospective cohort study conducted at Duke University Hospital (1996–2013) identified 51 cases: 14 in hematopoietic-cell transplant (HCT) recipients and 37 in solid-organ transplant (SOT) recipients. Infection occurred significantly earlier after transplantation in the HCT cohort (median 153 vs. 370 days). Pulmonary involvement was the predominant presentation in both groups. Overall outcomes were poor, with an attributable cure rate of only 29% among HCT recipients (46, 48).

**Figure 2:**
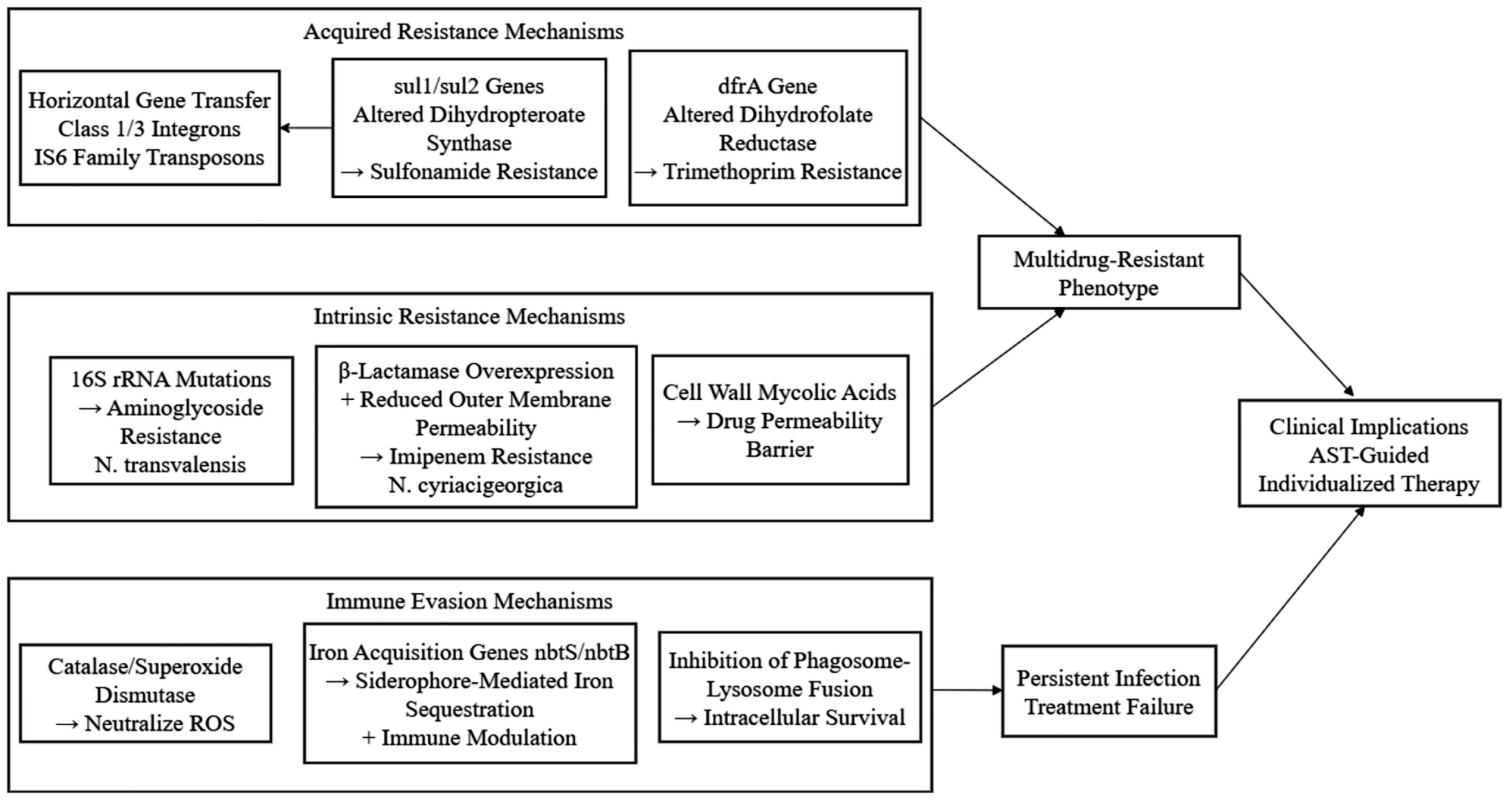
Molecular mechanisms of antimicrobial resistance in *Nocardia.*

Children with cystic fibrosis (CF) are likewise predisposed to *Nocardia* colonization and infection. A descriptive series of eight pediatric CF patients yielded molecularly confirmed isolates—including *N. farcinica*, *N. transvalensis*, and other species—that exhibited species-specific antimicrobial susceptibility patterns. Importantly, the detection of *Nocardia* in sputum did not invariably indicate active disease; thus, therapeutic decisions must be individualized after careful evaluation of microbiological, radiological, and clinical findings (23, 49). In addition, patients receiving prolonged immunosuppressive or corticosteroid therapy—such as those with autoimmune disorders or malignant neoplasms—show a markedly elevated risk of acquiring *Nocardia* infection (50). Well-documented cases of nocardiosis have been described in patients with rheumatoid arthritis, systemic lupus erythematosus, and other autoimmune disorders while they are receiving immunosuppressive regimens. Likewise, individuals with chronic pulmonary conditions—such as chronic obstructive pulmonary disease or bronchiectasis—are rendered vulnerable to *Nocardia* invasion because of structural and functional alterations in the lung parenchyma and airways (51, 52). Figure 2 systematically visualizes the molecular mechanisms underlying drug resistance and immune evasion in *Nocardia*, integrating acquired resistance (mediated by resistance genes and horizontal gene transfer), intrinsic resistance (driven by structural modifications and enzyme overexpression), and immune escape strategies (e.g., iron acquisition and ROS neutralization). This diagram clarifies the synergistic effects of these mechanisms in promoting treatment failure, and lays a theoretical foundation for the development of mechanism-based diagnostic targets and novel therapeutic strategies in subsequent chapters.

## 3 Pathological mechanisms of drug-resistant *Nocardia*

### 3.1 Genomic characteristics of drug-resistant *Nocardia*

Comprehensive genomic profiling of drug-resistant *Nocardia* is providing critical insight into the genetic basis of both antimicrobial resistance and pathogenicity. *N. farcinica* clinical isolate SZ1509 carries a plasmid-encoded IS6-composite transposon containing IS6100-linked *sul1*, terC, and four transcriptional regulators, driving constitutive *sul1* overexpression and high-level sulfamethoxazole resistance (MIC > 512 mg/L) (53). In 76 TMP-SMX-non-susceptible isolates (MIC ≥32:608 mg/L), *sul1* (93.4%), *sul2* (78.9%), and *dfrA(S1)* (14.7%) were the primary resistance determinants, with *blaTEM-1* and *blaZ* detected at 2.6% each (11). Class 1/3 integrons (93.42%/56.57% prevalence) serve as key platforms for resistance cassette capture and expression, while co-localization with transposons (IS6, IS6100) facilitates horizontal transfer between plasmids and chromosomes, accelerating interspecies resistance spread (54, 55).

Molecular surveys have established a tight genotype–phenotype correlation for sulfonamide resistance: *sul1* is present in 93.4% of resistant isolates (11). The gene encodes dihydropteroate synthase (DHPS), and structural alteration of this enzyme—via either active-site amino acid substitutions or post-translational modification—directly abolishes sulfamethoxazole binding, thereby conferring high-level resistance. Specifically, these molecular alterations induce conformational or binding-site changes in dihydropteroate synthase that prevent sulfonamides from occupying the p-aminobenzoic acid pocket, rendering the enzyme refractory to competitive inhibition and thus endowing the bacterium with high-level sulfonamide resistance (56).

Likewise, *sul2*—detected in 78.9% of TMP-SMX-resistant isolates—encodes an alternative dihydropteroate synthase whose active-site architecture has diverged sufficiently to evade sulfonamide blockade, thereby conferring resistance (11). Concurrently, the *dfrA(S1)* allele (a class-A dihydrofolate reductase gene) specifies a DHFR variant whose affinity for trimethoprim is drastically reduced. Consequently, trimethoprim fails to occupy the dihydrofolate-binding cleft, leaving the enzyme fully functional and rendering the organism resistant to the complete TMP-SMX combination (57, 58).

Recent pangenomic analyses of the genus *Nocardia* have revealed that resistance determinants can disseminate rapidly across species boundaries via horizontal gene transfer, underscoring the dynamic nature of the resistome in this taxon (59). In this process, class 1 and class 3 integrons—detected in 93.42 and 56.57% of TMP-SMX-resistant *Nocardia*, respectively—serve as the primary capture platforms: they incorporate *sul1*, *dfrA(S1)* and other resistance determinants into mobile gene cassettes that can be disseminated across species boundaries (11). Concomitantly, composite transposons such as IS6 carry *sul1* and jump between chromosomal loci and plasmids, further accelerating the intrageneric spread of resistance and continually expanding the *Nocardia* resistome (53).

The genomic architecture of *Nocardia* species is markedly heterogeneous. The *N. otitidiscaviarum* type strain UTF1, for example, encompasses a single 8,121,733-bp chromosome with 68.1% G + C content that is predicted to encode 7,697 proteins. In addition to a full repertoire of intrinsic antibiotic-resistance determinants, the genome harbors virulence homologs implicated in host-cell invasion, phagosome maturation arrest and intramacrophage survival—functions previously characterized in pathogenic mycobacteria and clinical *Nocardia* isolates (60). By contrast, the 8.2-Mb genome of *N. cerradoensis* carries 8,329 coding sequences and is enriched in innate resistance genes, polyketide- and non-ribosomal-peptide biosynthetic clusters, canonical virulence factors and multiple prophage regions, underscoring species-specific strategies for environmental persistence and pathogenicity (61). These inter-species genomic differences underpin the variable antimicrobial resistance profiles and virulence potentials observed among *Nocardia* taxa, thereby providing a rational genomic framework for the development of species-specific therapeutics and precision treatment strategies.

### 3.2 Mechanistic insights into drug resistance in *Nocardia*

The molecular basis of *Nocardia* resistance is multifaceted. At the genetic level, 93.42 and 78.94% of isolates non-susceptible to TMP-SMX harbor *sul1* and *sul2*, respectively—frequencies that underscore the central role of these genes in sulfonamide resistance (11). Table 2 clarifies the major drug resistance genes of *Nocardia*, their corresponding resistance phenotypes, associated species and global clinical prevalence, which is the molecular basis for resistance mechanism research and diagnostic target development. Concurrently, *Nocardia* spp. evade folate-pathway antagonists by acquiring mutations that remodel the target enzymes themselves. In a panel of eight *N. asteroides* and two *N. cyriacigeorgica* isolates, resistance to TMP-SMX was attributable to amino acid substitutions clustered around the active sites of dihydrofolate reductase (DHFR) and dihydropteroate synthase (DHPS), thereby reducing drug binding without compromising enzymatic function. Additionally, a previously uncharacterized paralogue of DHPS—designated DHPS2 or FolP2—has been found to acquire resistance-conferring mutations. The gene is co-transcribed with a downstream glucosyl-3-phosphoglycerate synthase, implying that the resulting putative bifunctional enzyme may contribute to sulfamethoxazole resistance by an as-yet-undefined mechanism (13).

**Table 2:** Major resistance genes and their clinical prevalence.

Resistance gene	Resistance phenotype	Associated *Nocardia* species	Region	Prevalence	References
*sul1*	Sulfonamide (e.g., TMP-SMX) resistance	TMP-SMX-resistant strains (*N. farcinica*, *N. brasiliensis*, etc.)	Multiple global regions	93.4% in TMP-SMX non-susceptible isolates	(11, 53)
*sul2*	Sulfonamide (e.g., TMP-SMX) resistance	TMP-SMX-resistant strains (*N. farcinica*, *N. brasiliensis*, etc.)	Multiple global regions	78.9% in TMP-SMX non-susceptible isolates	(11)
*dfrA(S1)*	TMP-SMX combination resistance	TMP-SMX-resistant strains (*N. farcinica*, *N. brasiliensis*, etc.)	Multiple global regions	14.7% in TMP-SMX non-susceptible isolates	(11)
*blaTEM-1*	β-lactam resistance	TMP-SMX-non-susceptible strains	Multiple global regions	2.6% in TMP-SMX non-susceptible isolates	(11)
*blaZ*	β-lactam resistance	TMP-SMX-non-susceptible strains	Multiple global regions	2.6% in TMP-SMX non-susceptible isolates	(11)

Species-specific resistance is a defining feature: *N. transvalensis* exhibits intrinsic aminoglycoside resistance via 16S rRNA gene mutations altering the 30S ribosomal subunit binding site (34), while most pathogenic species retain 95–99% amikacin susceptibility (4, 11). Carbapenem susceptibility varies markedly: *N. otitidiscaviarum* shows 16.67% imipenem susceptibility in China, whereas *N. farcinica* exhibits >90% susceptibility (62); an Australian multicenter study reported 59% imipenem non-susceptibility, with *N. cyriacigeorgica* displaying high-level resistance (MIC >16 mg/L) due to *β*-lactamase overexpression and reduced outer membrane permeability (63).

This mechanistic heterogeneity—rooted in genomic divergence and horizontal gene transfer—explains the failure of one-size-fits-all empirical therapy and highlights a critical translational gap: while resistance mechanisms are increasingly characterized, their integration into rapid diagnostic workflows (e.g., resistance gene panels for MALDI-TOF MS/NGS) remains underdeveloped. This gap mandates not only precise species identification and susceptibility testing but also the development of mechanism-based diagnostic tools to guide real-time therapeutic decisions (25).

### 3.3 Immune evasion strategies of *Nocardia* spp.

Human-pathogenic *Nocardia* (*N. farcinica*, *N. asteroides*, *N. nova*) employ species-specific immune evasion via cell wall components and secreted virulence factors: (1) Short-chain mycolic acids (conserved in pathogenic actinomycetes including *Nocardia*) hijack Coronin-1A to block macrophage autophagic fluxPubMed (64); (2) Nocardomycolic acids (major cell wall lipids of pathogenic *Nocardia*) inhibit phagosome-lysosome fusion and phagosomal maturation (65); (3) Secreted and surface-associated superoxide dismutase (SOD) and catalase neutralize phagocyte-derived reactive oxygen species (ROS), abrogating oxidative killing (66) (4) *Nocardia* produce siderophores (e.g., nocobactins, nocardichelins) with potent metal-chelating activity, which sequester host iron to support bacterial intracellular survival (67, 68).

Piscine nocardiosis is primarily caused by *Nocardia seriolae*, a phylogenetically distinct species whose immune evasion relies on host-specific virulence factors (e.g., mycolic acid-rich cell walls, GapA adhesin) adapted to teleost immune systems (69, 70). However, its molecular strategies are fundamentally different from those of human-pathogenic *Nocardia*, and insights from piscine models cannot be extrapolated to human infections (71). In human nocardiosis, immune evasion is well characterized in key pathogenic species (*Nocardia farcinica*, *Nocardia nova* complex, *Nocardia asteroides*). These strains secrete catalase and superoxide dismutase (SOD) that synergistically neutralize phagocyte-derived ROS, dismantling the oxidative killing machinery (72, 73). Iron-acquisition genes (*nbtB*, *nbtS*) are also pivotal: they enable siderophore-mediated iron sequestration, activate host inflammatory cascades (via NF-κB/MAPK signaling), and induce immune cell apoptosis, promoting persistent infection (74, 75). Clinical data link elevated expression of iron-acquisition genes to longer abscess-resolution times and higher treatment failure rates in pulmonary nocardiosis, confirming their role in disease severity (75).

In human nocardiosis, immune evasion is well characterized in key pathogenic species (*Nocardia farcinica*, *Nocardia nova* complex, *Nocardia asteroides*). These strains produce catalase and superoxide dismutase (SOD) that cooperatively neutralize phagocyte-derived reactive oxygen species (ROS), thereby resisting oxidative killing (67, 76, 77). Iron-acquisition genes (*nbtB*, *nbtS*) are also pivotal: they mediate siderophore-dependent iron sequestration, activate host inflammatory cascades via NF-κB/MAPK signaling, and induce immune cell apoptosis, thereby facilitating persistent infection (74, 78). Clinical data associate elevated expression of iron-acquisition genes with delayed abscess resolution and higher rates of treatment failure in pulmonary nocardiosis, supporting their contribution to disease severity (2, 5, 79).

Analysis of clinical specimens from patients with nocardiosis has further disclosed a direct link between iron-acquisition genes and disease progression. Among these, *nbtB* and *nbtS* have been validated as pivotal virulence determinants: both are indispensable for efficient iron uptake and full bacterial fitness. Notably, deletion of *nbtS* alone completely abolishes the neurological manifestations otherwise elicited by *Nocardia farcinica*, underscoring the centrality of siderophore-mediated iron acquisition in the pathogenesis of invasive *Nocardia* disease (74). In patients with pulmonary nocardiosis, transcript levels of iron-acquisition genes differ markedly between those who develop lung abscesses and those who do not; moreover, the magnitude of expression correlates positively with the radiological extent of pulmonary lesions, thereby serving as an indirect index of disease progression (74, 80).

Mechanistic analyses have revealed that the *nbtS*-encoded protein functions as a pivotal virulence factor: its expression triggers host inflammatory cascades through the activation of specific signaling pathways. Concurrently, the bacterium’s iron-acquisition genes enable the synthesis of siderophores that compete for and sequester host-free iron, thereby satisfying the pathogen’s metabolic requirements while simultaneously depriving the host of this essential nutrient (78). This siderophore-driven iron acquisition not only facilitates bacterial colonization and proliferation within host tissues but also orchestrates a dynamic pro-inflammatory response. Following *Nocardia* infection, host levels of TNF-*α* and IL-6 rise rapidly, and the *nbtS*-encoded protein markedly amplifies TNF-α production. Activation of the associated signaling cascade concurrently triggers apoptosis in host immune cells, thereby attenuating immune-mediated clearance and promoting persistent infection (75). Clinical investigations demonstrate that pulmonary nocardiosis patients with elevated expression of iron-acquisition genes exhibit significantly longer abscess-resolution times and a higher incidence of treatment failure compared with those displaying low expression. These *ex vivo* data establish a direct correlation between transcriptional up-regulation of iron-uptake loci and disease severity, consolidating the concept that iron-scavenging regulation constitutes a central mechanism underlying *Nocardia* immune evasion and pathogenicity (81). Collectively, these human-specific immune evasion mechanisms—coupled with resistance gene dissemination—create a dual barrier to treatment success: pathogens not only evade host immunity but also rapidly acquire drug resistance. The divergence from piscine *Nocardia seriolae* underscores the limitations of non-human models, highlighting a key knowledge gap: human-relevant *in vitro/in vivo* models (e.g., organoids, immunocompromised mouse co-infection models) are urgently needed to validate virulence targets (e.g., NbtS, MycP) and optimize novel therapies, bridging the gap between molecular mechanistic insights and clinical efficacy.

## 4 Diagnostic technologies for drug-resistant *Nocardia*

Research into the pathogenic mechanisms of drug-resistant *Nocardia* has provided pivotal molecular targets for the development and clinical application of novel diagnostic techniques. Resistance in these organisms is primarily attributable to specific genetic alterations: missense mutations within the DHFR gene family confer resistance to TMP-SMX, whereas sulfonamide resistance is similarly driven by sequence variants in associated resistance determinants (12). Moreover, both resistance profiles and virulence-gene repertoires differ markedly among species and even among strains. *Nocardia cyriacigeorgica* alone, for instance, segregates into five major phylogenetic clades whose complements of resistance and virulence genes vary extensively, with core genes accounting for only 3.4% of the pangenome (82). Additionally, *Nocardia* species evade host immunity by secreting catalase-related proteins, which blunts the oxidative burst and renders early infection paucisymptomatic; consequently, standard culture frequently misses the pathogen and delays detection. These biological features impose three simultaneous requirements on the clinical laboratory: (i) rapid speciation, (ii) accurate mapping of resistance determinants, and (iii) minimization of both false-negative and false-positive results. Conventional culture remains the reference for species-level identification, yet it demands 3–14 days of incubation and, for some isolates, up to 4–6 weeks (83), while offering no information on resistance genes—an information gap that is incompatible with the urgency of mechanism-guided therapy. Integrating molecular assays and rapid phenotypic tests into a single “mechanism-oriented, technology-complementary” diagnostic platform is therefore imperative.

### 4.1 Clinical criteria for the diagnosis of drug-resistant *Nocardia* infection

Accurate diagnosis of drug-resistant *Nocardia* infection requires a comprehensive, multi-factorial approach. Clinically, nocardiosis can present as pulmonary, cutaneous-soft-tissue, or central-nervous-system (CNS) disease. Pulmonary involvement typically produces cough, sputum production and fever, and computed-tomography or plain radiographs may reveal nodules, cavitation, or infiltrates (4). Cutaneous-soft-tissue infections manifest as abscesses, ulceration or cellulitis, whereas CNS invasion may precipitate headache, focal neurologic deficits or altered mental status (84).

Laboratory confirmation is pivotal. Microbial culture is the clinical gold standard for *Nocardia* isolation and phenotypic susceptibility testing (57, 81), requiring 5–14 days of 35–37 °C aerobic incubation on enriched media (brain-heart-infusion agar, Columbia blood agar with 5% sheep blood); fastidious isolates may need up to 21 days (85, 86). Selective media (modified Thayer-Martin agar) improve recovery rates by 30–40% via commensal flora inhibition.

Antimicrobial susceptibility testing is equally indispensable: quantitative determination of the isolate’s in-vitro response to clinically relevant agents permits selection of an evidence-based, pathogen-tailored therapeutic regimen (87). For example, a broth-microdilution survey of 52 Pakistani isolates revealed that 90.4% were susceptible to TMP-SMX, 38.5% to imipenem, 94.2% to amikacin, and 100% to linezolid (88). Additionally, molecular methods—particularly 16S rRNA gene sequencing—enable rapid and accurate speciation, routinely achieving ≥ 98% DNA homology with reference *Nocardia* strains, thereby providing the clinician with high-resolution information that guides both diagnosis and therapy (89).

### 4.2 Molecular diagnostic approaches for drug-resistant *Nocardia*

Molecular diagnostic methods are pivotal for the identification of drug-resistant *Nocardia*. Sequencing of the 16S rRNA gene enables reliable species-level assignment; in a Brazilian study, a 1,491-bp segment of this locus was used to characterize seven clinical isolates as *Nocardia farcinica* (*n* = 4), *Nocardia nova* (*n* = 2), and *Nocardia asiatica* (*n* = 1). Notably, 43% of these strains exhibited resistance to both gentamicin and TMP-SMX (38). Multilocus sequence analysis (MLSA) that incorporates partial sequences of *gyrB*, *secA1*, and *hsp65* raises the accuracy of *Nocardia* identification to 99.5% (90). A recent Iranian meta-analysis identified *Nocardia asteroides* (21%), *Nocardia cyriacigeorgica* (17%), and *Nocardia farcinica* (10%) as the predominant agents of human nocardiosis in the region, with marked inter-species differences in antimicrobial susceptibility profiles (91).

Molecular diagnostics also enable direct detection of resistance determinants. In a survey of 76 trimethoprim-sulfamethoxazole-resistant *Nocardia* isolates (MIC ≥ 32:608 mg/L), PCR followed by sequencing revealed *sul1* in 93.4% of strains, *sul2* in 78.9%, and *dfrA(S1)* in 14.7%—genotypic data that provide a precise rationale for antimicrobial stewardship (11). Matrix-assisted laser desorption/ionization time-of-flight mass spectrometry (MALDI-TOF MS) has also been validated for rapid and accurate identification of *Nocardia* spp. (92). A prospective study conducted in Henan, China, between 2017 and 2023 combined MALDI-TOF MS with 16S rRNA gene sequencing to achieve unequivocal species-level identification of 71 clinical *Nocardia* isolates, whose antimicrobial susceptibility profiles were subsequently determined by broth microdilution (93). The integrated deployment of these molecular approaches has elevated the accuracy of *Nocardia* species identification to near-definitive levels, substantially enhancing both the precision and the speed of nocardiosis diagnostics.

### 4.3 Rapid detection technologies for drug-resistant *Nocardia*

Rapid detection technologies are indispensable for the timely diagnosis of drug-resistant *Nocardia* infections. Available platforms differ markedly in cost-effectiveness, intended use-case, and analytical performance, so the choice must be tailored to the level of the health-care facility and the clinical urgency. --Conventional culture is the most cost-effective modality (≈120–180 RMB/test) and enables broth microdilution-based MIC determination, but requires 7–21 days for combined isolation/susceptibility testing CLSI. Unlike molecular methods, culture only reports phenotypic resistance without identifying genetic determinants [*sul1*, *dfrA(S1)*] PubMed, making it the primary approach in resource-limited settings and a confirmatory tool in tertiary centers (94–96).

MALDI-TOF MS offers a cost-controllable solution: after in-house expansion and curation of the reference library, the unit price per test drops markedly, and a 5-year cohort of 153 *Nocardia* isolates showed a cumulative saving of ≈ 46,000 RMB. With the customized library, the rate of correct species-level identification reached 91.5% (97). MALDI-TOF MS shortens time-to-identification by an average of 1.45 days compared with conventional culture. When coupled with optimized media and a continuously updated database, the correct-identification rate for commonly encountered *Nocardia* spp. rises to 93.0%, and in-house libraries refined with additional reference spectra have achieved 98.7% accuracy (98, 99). MALDI-TOF MS therefore provides same-day speciation and is well suited for routine use in tertiary-level hospitals with moderate-to-high diagnostic throughput. Its reliability falls sharply, however, when bacterial density is < 5.0 × 10^3^ CFU mL^−1^. Next-generation sequencing (NGS) incurs a higher cost (≈ 3,600–4,800 RMB per run) but can deliver both species identification and prediction of antibiotic susceptibility within 48 h, making it the method of choice for critically ill patients—such as those with CNS nocardiosis—or for otherwise unresolved cases in tertiary referral centers (100, 101). A simplified multiplex PCR assay that targets conserved regions of the *Nocardia* 16S rRNA gene is markedly less expensive than NGS, returns results within 3 h, and requires only a basic thermocycler—features that make it the preferred rapid-screening tool for primary-level hospitals. The analytical sensitivity reaches 3 × 10^2^ copies mL^−1^, sufficient for early clinical rule-in or rule-out (102, 103).

False-positive and false-negative rates vary with specimen type. In NGS-based detection of *Nocardia*, sputum is prone to environmental contamination, yielding lower specificity than cerebrospinal fluid (CSF). CSF, being largely free of background flora, offers higher specificity, yet its low bacterial load can produce false negatives. Skin-pus samples are compromised by commensal cutaneous microbiota, reducing accuracy. Consequently, results must always be interpreted in conjunction with clinical findings (104, 105). When MALDI-TOF MS is applied to pure *Nocardia* cultures, the false-positive rate is ~0.8% (106); direct analysis of clinical specimens such as sputum raises this figure to 2.4–3.4% (107), depending on the culture medium used. The overall mis-identification rate for *Nocardia* is ~3%, and false-negative results are primarily linked to the completeness of the reference library and the sample-processing protocol (108). Conventional culture exhibits a low false-positive rate of 0.5–1.0%, yet its false-negative rate is unacceptably high: on standard media up to 60% of *Nocardia* infections are missed, primarily because the organism grows slowly and can be readily overgrown by contaminating flora (109). These data underscore a critical systemic challenge: diagnostic technologies exhibit trade-offs in sensitivity, cost, and turnaround time that create care disparities. Conventional culture is widely accessible but misses 40–60% of infections, while NGS enables rapid resistance prediction but is unaffordable in low-resource settings (110). MALDI-TOF MS balances cost and performance, with 84.2–98.7% species identification accuracy for pure cultures (15), but becomes unreliable at bacterial densities below 5.0 × 10^3^ CFU/mL. This heterogeneity mandates a tiered diagnostic algorithm: multiplex PCR (3-h turnaround, 3 × 10^2^ copies/mL sensitivity) for primary care screening (111), MALDI-TOF MS for routine use in tertiary centers, and NGS for critically ill patients, thereby balancing accessibility and diagnostic rigor. Implementation is currently hindered by two key gaps: the lack of low-cost point-of-care testing kits for primary care and standardized bioinformatic pipelines for NGS-based resistance prediction. Closing these gaps could reduce diagnostic delays by 50–70% and improve clinical outcomes (112). Table 3 summarizes the core performance parameters, including sensitivity, specificity, cost and turnaround time, of mainstream diagnostic technologies for drug-resistant *Nocardia*, providing a reference for clinical selection based on institutional conditions and clinical urgency.

**Table 3:** Comparison of sensitivity, specificity, cost, and turnaround time of different diagnostic technologies.

Diagnostic technology	Sensitivity	Specificity	Cost	Turnaround time	References
Conventional culture	Approximately 40–60% (high false-negative rate due to easy overgrowth by contaminating flora)	99.0–99.5% (false-positive rate: 0.5–1.0%)	≈153.6 RMB/test	3–14 days, some strains require up to 4–6 weeks	(23, 188)
MALDI-TOF MS (pure culture)	91.5–98.7% for common Nocardia species	99.2% (false-positive rate: ≈0.8%)	Cumulative savings of ≈46,000 RMB in a 5-year cohort of 153 Nocardia isolates	1.45 days shorter than conventional culture on average; same-day identification for pure cultures	(64, 69, 196)
MALDI-TOF MS (direct clinical specimen, e.g., sputum)	Not separately reported, affected by specimen type	96.6–97.6% (false-positive rate: 2.4–3.4%)	Same as for pure culture testing	Same as for pure culture testing	(70)
NGS	Not separately reported; capable of detecting low-load pathogens	Higher specificity for CSF; lower specificity for sputum due to environmental contamination	≈3,600–4,800 RMB/run	Species identification and antibiotic susceptibility prediction within 48 h	(65, 171, 189)
Multiplex PCR	Analytical sensitivity: 3 × 10^2^ copies/mL	Not separately reported; results need to be interpreted in conjunction with clinical findings	Lower than NGS	Results available within 3 h	(67)

**Figure 3:**
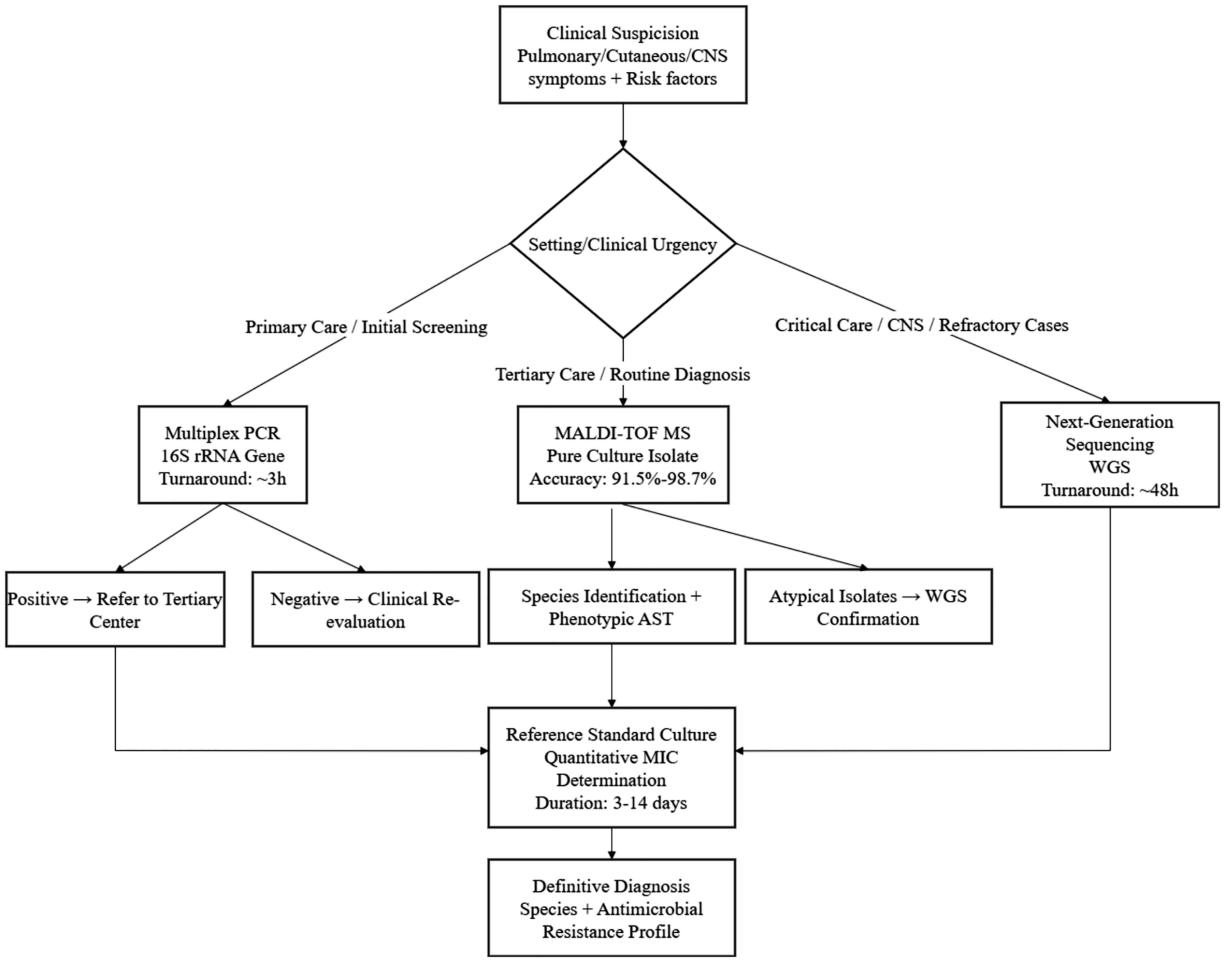
Diagnostic workflow for drug-resistant *Nocardia* infections.

To intuitively integrate the diagnostic principles, technical characteristics, and clinical application scenarios of drug-resistant *Nocardia* infections summarized in this chapter, Figure 3 constructs a tiered diagnostic workflow. This workflow prioritizes accessibility and diagnostic rigor, matching different detection technologies (multiplex PCR, MALDI-TOF MS, NGS, conventional culture) to the capabilities of primary, secondary, and tertiary medical institutions. It also clarifies the selection logic based on clinical urgency and specimen types, providing a practical operational framework for clinicians to rapidly and accurately diagnose drug-resistant *Nocardia* infections.

## 5 Treatment strategies for infections caused by drug-resistant *Nocardia*

### 5.1 Antibiotic options for drug-resistant *Nocardia*

Rational antibiotic selection is the cornerstone of managing infections caused by drug-resistant *Nocardia*, with significant regional variability in antimicrobial activity that must guide clinical decision-making. Linezolid (oxazolidinone targeting 50S ribosomal subunit assembly) is a backbone for drug-resistant *Nocardia* infections, with near-100% global susceptibility and excellent blood–brain barrier penetration (CSF concentration 30–50% of serum), making it first-line for CNS nocardiosis (113, 114). TMP-SMX remains widely used, but its resistance rate varies dramatically by region: 2% in U. S. multicenter data (2005–2011) (115), 11% in Australia (25), and 1.1–2.0% in China (4), reflecting differences in antimicrobial use (e.g., TMP-SMX prophylaxis in transplant populations). This regional heterogeneity highlights the need for AST prior to empirical therapy. Notably, species-specific heterogeneity is common, with several taxa displaying elevated MICs (78), and a recent analysis of 76 TMP-SMX-resistant isolates identified diverse resistance determinants [*sul1*, *sul2*, *dfrA(S1)*], underscoring the need for susceptibility testing (11).

*Nocardia*: Amikacin retains potent activity globally but with regional variations: China’s 13-year nationwide survey reported 99.3% susceptibility (18). While Greek isolates showed 96.4% susceptibility (116), and Australian strains had 95–99% susceptibility (11). In contrast, 64% of *Nocardia* isolates from keratitis cases in the Americas were non-susceptible to amikacin, with N. amikacinitolerans accounting for 73.3% of resistant strains (14).

Other agents exhibit striking regional differences reflective of antimicrobial use patterns: Ceftriaxone resistance is 11.5% in Kenya (117)_,_ 8.3% in India (118), and 3.2% in China (5); clarithromycin resistance reaches 26.9% in Kenya and 18.4% in Nigeria (63), but only 4.5% in Australia (4). Gentamicin retains 100% susceptibility in Kenya and 98.7% in Brazil (119), but drops to 89.2% in Vietnam (120). Imipenem susceptibility ranges from 41.2% in Nigeria (121) and 42.9% in Greece (116) to 40.7% in Sydney, Australia (122) and 76.9–88.9% in China (5). Ciprofloxacin shows 98.1% susceptibility in Kenya (117)_,_ but only <30% susceptibility in Latin American mycetoma isolates (Argentina, representative of regional resistance), highlighting the need for region-specific empirical therapy guidelines (123, 124).

*Nocardia farcinica* exhibits uniform resistance to ceftriaxone (125), while the *Nocardia asteroides* complex shows heterogeneous susceptibility (57% susceptible among Kuwaiti soil isolates, up to 82% in some clinical collections) (8, 126). *Nocardia brasiliensis* has a 51% multidrug-resistance rate, with nemonoxacin demonstrating superior activity among fluoroquinolones compared to ciprofloxacin (115, 125, 127). Therefore, antimicrobial selection requires a precision medicine approach that integrates three interconnected pillars: species-specific resistance profiles, regional antimicrobial use patterns (which drive resistance dissemination), and patient-specific factors (comorbidities, pharmacokinetics). A key unresolved gap is the lack of large-scale prospective data linking genotype [e.g., *sul1*, *dfrA(S1)*] to treatment outcomes, which limits the ability to translate molecular resistance data into clinical decision-making. Filling this gap would enable mechanism-based therapy (e.g., avoiding TMP-SMX in *sul1*-positive isolates) rather than reliance on empirical regimens, reducing treatment failure rates.

### 5.2 Individualized treatment regimens for drug-resistant *Nocardia*

Tailored regimens are pivotal for optimizing outcomes in infections caused by drug-resistant *Nocardia*. Doses must be adjusted according to the pharmacokinetic properties of each agent in special populations—e.g., patients with hepatic or renal impairment, pregnant women—while vigilantly monitoring for synergistic adverse-event risks inherent to combination therapy.

#### 5.2.1 Dose adjustment in patients with hepatic and renal impairment

Amikacin (aminoglycoside targeting 30S ribosomal subunit) is ≥90% renally excreted, requiring CrCl-based dosing to avoid nephrotoxicity/ototoxicity (128). Adults with CrCl ≥50 mL/min: 7.5 mg/kg IV 12 h (15 mg/kg/day) (128); CrCl 30–49 mL/min: 7.5 mg/kg 18 h; CrCl 10–29 mL/min: 7.5 mg/kg 24 h; ESRD (CrCl <10 mL/min): 5 mg/kg 48–72 h (129). Mandatory TDM: target peak 20.0–35.0 μg/mL (30 min post-infusion), trough 1–4 μg/mL (130, 131). In kidney-transplant recipients with nocardiosis and reduced CrCl (e.g., 25 mL/min), amikacin 5.5 mg/kg once every 24 h or an individualized extended-interval regimen is a reasonable option to limit nephrotoxicity while maintaining infection control (130, 131).

Linezolid: No dose adjustment is required in mild-to-moderate hepatic impairment (Child-Pugh A or B); in severe impairment (Child-Pugh C), the standard 600 mg every 12 h should be reduced to 600 mg once daily, with enhanced hematological monitoring (132, 133). No dosage adjustment of linezolid is necessary in patients with renal impairment, including those with end-stage renal disease on dialysis; however, these individuals carry a higher risk of thrombocytopenia, so platelet counts must be monitored to avert potential myelosuppression (134, 135).

#### 5.2.2 Drug selection in pregnant patients

In pregnant women with nocardiosis, the benefit–risk balance between maternal efficacy and fetal safety must be carefully weighed. During the first trimester, TMP-SMX may raise the incidence of fetal neural-tube defects to 0.7% and should therefore be reserved for situations in which the anticipated benefit clearly outweighs this risk; concomitant folic-acid supplementation and specialist counseling are mandatory (119). In the second and third trimesters, TMP-SMX may be used cautiously, yet the fetal risks of urogenital and cardiovascular malformations remain elevated; maternal blood counts and fetal ultrasonography must therefore be monitored (121). Linezolid was not teratogenic in animal studies, but human data are scarce. It should be reserved for pregnant women who have failed or are intolerant to other agents. The recommended dose is 600 mg every 12 h; duration must be individualized according to infection control to minimize fetal exposure to myelosuppression associated with prolonged therapy, and maternal hematologic parameters must be monitored throughout treatment (133). Amikacin carries a 3–5% risk of fetal ototoxicity and should be restricted to severe infections when no alternatives exist. In pregnant women with normal renal function, the recommended dose is 7.5 mg/kg once every 24 h; maternal serum drug levels and fetal development must be closely monitored (136, 137).

#### 5.2.3 Synergistic risk of adverse drug reactions in polypharmacy

Both co-trimoxazole and amikacin can cause nephrotoxicity, and their concurrent use produces an additive risk. Their combination has been identified as an independent risk factor for acute kidney injury; therefore, renal function must be rigorously monitored whenever the two drugs are administered together (120).

Linezolid can induce thrombocytopenia, whereas carbapenems may cause leukopenia; combined use markedly increases the risk of severe thrombocytopenia, and this combination is a significant risk factor for linezolid-related severe platelet reduction. It is recommended that complete blood counts be monitored more frequently during therapy; if the platelet count falls below 75 × 10^9^/L, the linezolid dose should be reduced or an alternative agent substituted (138, 139).

#### 5.2.4 Therapeutic strategies for special populations

In immunocompromised patients—such as transplant recipients or individuals on long-term immunosuppressants—nocardiosis is both more common and potentially more severe, mandating heightened therapeutic vigilance. In a cohort of transplant recipients, management was individualized according to disease extent, antimicrobial susceptibility, and hepatic/renal function. Regimens included co-trimoxazole, renally adjusted amikacin, or linezolid; selected cases were supplemented with surgical intervention, notably aspiration or resection of cerebral abscesses (140).

In pediatric patients with cystic fibrosis who develop *Nocardia* infection, antimicrobial selection must be guided by the identified species and its susceptibility profile. For example, if *Nocardia farcinica* is isolated and demonstrates resistance to gentamicin, tobramycin, and cefotaxime but susceptibility to co-trimoxazole and amikacin, then the use of resistant agents should be avoided in the treatment regimen (23). Owing to the immaturity of renal function in children, amikacin dosage must be individualized according to age, body weight, and disease severity. The conventional daily dose is 15–20 mg/kg (141); For neonates >2 kg and >7 days post-partum, 10 mg/kg every 8 h (total 30 mg/kg/day) may be used. Audiometry should be performed periodically, and serum drug concentrations should be monitored to refine dosing and minimize ototoxicity (142).

Underlying diseases influence the treatment regimen: in patients with chronic pulmonary diseases (e.g., COPD, bronchiectasis), the duration of antibiotic therapy should be prolonged (a 14-day course is recommended), and attention must be paid to the impact of drugs on pulmonary function. Medications that may induce pulmonary fibrosis—such as bleomycin, methotrexate, and cyclophosphamide—should be avoided (143, 144).

### 5.3 Combination antimicrobial strategies for drug-resistant *Nocardia* infections

Table 4 systematically lists the evidence-based first-line and alternative treatment regimens for drug-resistant *Nocardia* infections, which are stratified by major pathogenic species, infection sites and patient immune status, and attached with clear dosage adjustment principles. Combination therapy is an important strategy for managing drug-resistant *Nocardia* infections (145). Owing to the intrinsic resistance of *Nocardia* spp., monotherapy frequently yields suboptimal outcomes. In pulmonary nocardiosis, co-trimoxazole is therefore commonly combined with additional agents. In several studies, patients with severe disease or a high risk of resistance were treated with co-trimoxazole plus amikacin or linezolid, and this strategy achieved favorable therapeutic results (146).

**Table 4:** First-line and alternative treatment regimens based on species, infection site, and patient status.

Major pathogenic *Nocardia* species	Infection site	Patient immune status	First-line treatment regimen	Alternative treatment regimen	Principles of dosage adjustment	References
*N. farcinica*	Lung	Immunocompetent	TMP-SMX + Amikacin	Linezolid + Imipenem	1. Amikacin: Adjust by CrCl; monitor serum concentrations (trough: 1–4 μg/mL; peak: 20.0–35.0 μg/mL).	(4, 8, 73, 190)
					2. Linezolid: No renal adjustment; 600 mg qd for severe hepatic impairment (Child-Pugh C) with hematological monitoring.	
*N. farcinica*	Lung	Immunocompromised (e.g., transplant recipients, long-term immunosuppressant users)	Linezolid + Amikacin	Tigecycline + Imipenem	1. Amikacin: Individualized dose for transplant recipients with reduced CrCl.	(16, 73, 87)
					2. Linezolid: Regular platelet count monitoring.	
*N. farcinica*	Central Nervous System	All immune statuses	Linezolid + Imipenem	Amikacin + Ceftriaxone (note strain resistance)	1. Imipenem: Adjust by renal function; increase dose for severe infections.	(102, 191)
					2. Amikacin: Ensure blood–brain barrier penetration; monitor serum concentrations.	
*N. brasiliensis*	Cutaneous-Soft Tissue	Immunocompetent	TMP-SMX	Linezolid + Minocycline	1. TMP-SMX: Adult 160/800 mg q12h; adjust by weight.	(78, 103)
					2. Linezolid: Adult 600 mg q12h; weight-based dosage for children.	
*N. brasiliensis*	Cutaneous-Soft Tissue	Immunocompromised	Linezolid + Amikacin	Tigecycline + Cefepime	1. Amikacin: CrCl-based adjustment for renal impairment.	(78, 95)
					2. Tigecycline: Loading 100 mg, then 50 mg q12h; adjust for hepatic impairment.	
*N. cyriacigeorgica*	Lung	Immunocompetent	TMP-SMX + Amikacin	Linezolid + Moxifloxacin	1. Moxifloxacin: Adult 400 mg qd; no renal adjustment; caution in hepatic impairment.	(5, 18, 74)
					2. Amikacin: Monitor renal function during standard dosage.	
*N. cyriacigeorgica*	Lung	Pregnant (second and third trimesters)	Linezolid	Amikacin (restricted to severe infections with no alternatives)	1. Linezolid: 600 mg q12h; shorten treatment duration; monitor maternal hematology.	(85, 90, 197)
					2. Amikacin: 7.5 mg/kg qd; monitor maternal serum concentrations and fetal development.	
*N. otitidiscaviarum*	Lung	Immunocompetent	Linezolid + Amikacin	Tigecycline + Imipenem (note low strain susceptibility to imipenem)	1. Imipenem: Use per susceptibility results.	(31, 73)
					2. Amikacin: Standard dosage with renal function monitoring.	
*N. asteroides* complex	Peritoneum	Pediatric (peritoneal dialysis patients)	Linezolid	TMP-SMX (exclude if allergic)	1. Linezolid: Weight-based dosage for children; monitor platelets.	(105)
					2. TMP-SMX: Weight-based dosage to avoid overdose.	

**Figure 4:**
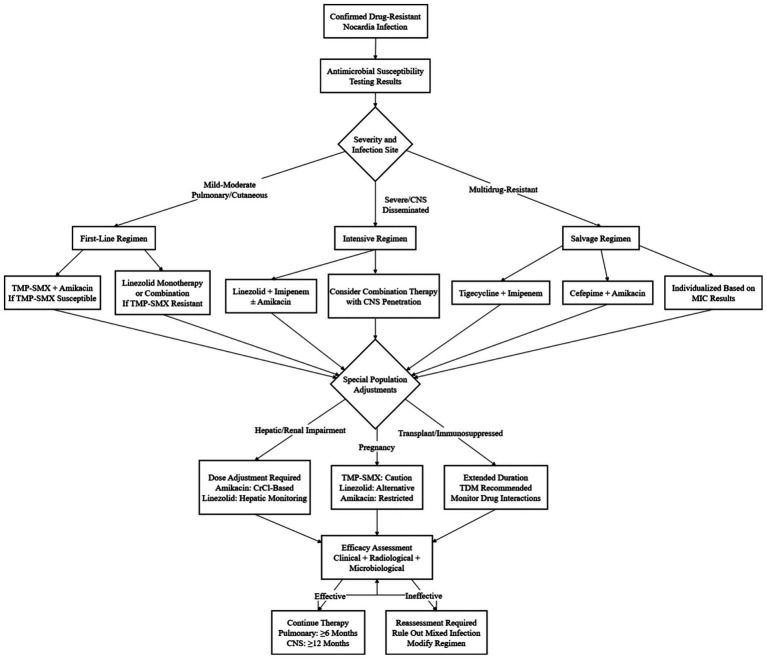
Treatment algorithm for drug-resistant *Nocardia* infections.

For central nervous system *Nocardia* infections, combination therapy is even more critical given the severity of disease and treatment challenges. In several cases, regimens employing carbapenems such as imipenem in conjunction with aminoglycosides or other antimicrobial agents have been utilized to enhance antibacterial efficacy (147). Moreover, combination therapy should be considered for skin and soft-tissue infections when antimicrobial resistance is documented. In the treatment of cutaneous abscesses caused by drug-resistant *Nocardia*, for example, linezolid combined with other antibiotics to which the isolate is susceptible can be administered concurrently with surgical debridement to accelerate infection control and wound healing (148). Combination therapy—when guided by susceptibility testing and mechanism of action—addresses the intrinsic limitations of monotherapy (e.g., resistance selection, suboptimal tissue penetration). However, a critical knowledge gap persists: the lack of evidence-based guidelines for synergistic combinations, particularly for multidrug-resistant strains and special populations (e.g., pregnant patients, ESRD). Prospective clinical trials comparing mechanism-based combinations (e.g., linezolid + amikacin targeting protein synthesis vs. imipenem + linezolid targeting cell wall/protein synthesis) are needed to define optimal regimens, moving beyond empirical use to rational, evidence-based combination therapy. To translate the evidence-based treatment principles, antibiotic selection, and individualized adjustment strategies detailed in this chapter into clinical practice, Figure 4 presents a standardized treatment algorithm for drug-resistant *Nocardia* infections. This algorithm comprehensively considers key factors such as pathogenic species, infection site, patient immune status, and comorbidities, guiding clinicians to rapidly match first-line/alternative regimens and dosage adjustment schemes. It serves as a bridge between theoretical knowledge and clinical operation, providing a concise and actionable tool for optimizing treatment outcomes.

## 6 Clinical practice in the treatment of drug-resistant *nocardiosis*

### 6.1 Clinical case analysis of drug-resistant *Nocardia* infections

Clinical case analyses contribute to a deeper understanding of the characteristics and management strategies of drug-resistant *Nocardia* infections. A 79-year-old man with myasthenia gravis on chronic corticosteroid therapy developed multiple leg abscesses caused by *Nocardia pseudobrasiliensis*, which exhibited resistance to multiple antimicrobial agents and complicated treatment. This case illustrates that the emergence of uncommon *Nocardia* species and their associated resistance profiles increases the complexity of clinical diagnosis and therapy (149). In another case, a 13-year-old girl undergoing peritoneal dialysis developed *Nocardia asteroides*-associated peritonitis with intraperitoneal abscesses; prolonged linezolid therapy eventually achieved disease control. This episode underscores both the diagnostic and therapeutic challenges posed by *Nocardia* infections and the difficulty of defining the optimal treatment duration (150).

Pulmonary infection has also been described in immunocompetent patients. A 61-year-old Japanese woman with bronchiectasis, for example, developed *Nocardia mexicana* pneumonia caused by a multidrug-resistant isolate that was successfully treated with biapenem, intravenous amikacin, and oral linezolid (151). Additionally, several cases have demonstrated co-infection with *Nocardia* and other pathogens, further complicating diagnosis and management. In a 32-year-old patient with Crohn’s disease, pulmonary co-infection with *Nocardia* spp. and *Mycobacterium avium* was documented; the subject required a multi-drug antibiotic regimen. Such observations remind clinicians that complicated infections demand a comprehensive, multipathogen-oriented therapeutic strategy (152).

### 6.2 Efficacy evaluation of treatments for drug-resistant *Nocardia* infections

Evaluation of therapeutic efficacy against drug-resistant *Nocardia* is essential for regimen adjustment and prognostication; *in vitro* antimicrobial susceptibility testing constitutes one of the key foundations for such assessment (23). Determination of the minimal inhibitory concentration (MIC) of various agents against *Nocardia* spp. quantifies their in-vitro potency. In a survey of 101 isolates, tedizolid exhibited lower MIC_90_ values than linezolid against the most prevalent species, indicating superior in-vitro activity and offering clinicians a data-driven option for therapy selection (153).

In clinical practice, therapeutic efficacy must be appraised by integrating microbiological data with symptomatic, physical, and radiological findings. In pulmonary nocardiosis, resolution of cough and fever together with radiographic improvement—shrinkage of nodules, closure of cavities—constitutes objective evidence of response. Numerous cases have documented progressive clinical amelioration and gradual radiological clearance after prolonged antibiotic courses, corroborating the adequacy of the chosen regimen (154, 155). Nevertheless, mortality occurs in 20–40% of patients (156), driven by the interplay of three interconnected factors: unrecognized multidrug resistance, impaired host immunity (e.g., transplant recipients), and suboptimal drug exposure (e.g., inadequate dosing in renal impairment). This highlights a critical translational gap: the lack of validated surrogate markers (e.g., resistance gene expression, cytokine profiles) to predict treatment response and guide early regimen adjustment. Integrating molecular monitoring (e.g., serial NGS of clinical specimens) with clinical/radiological assessments could enable personalized treatment duration and reduce relapse rates, particularly in high-risk populations (157).

### 6.3 Side-effect management in the treatment of drug-resistant *Nocardia* infections

During therapy for drug-resistant *Nocardia*, meticulous management of adverse drug effects is mandatory. Co-trimoxazole, a frequently employed agent, can elicit a spectrum of toxicities, including hypersensitivity reactions and hematologic adverse events. In some cases, patients developed severe hyponatremia while receiving co-trimoxazole, necessitating substitution of the drug (158). Although linezolid exhibits excellent antibacterial activity, prolonged administration may produce significant toxicity; in a renal-transplant patient treated for 4 months, severe sensory neuropathy developed, forcing discontinuation of linezolid and completion of therapy with azithromycin (159).

To minimize adverse effects, clinicians must tailor both agent and dose to the individual patient. In patients with documented sulfonamide hypersensitivity, co-trimoxazole is contraindicated and an alternative agent should be selected. Throughout linezolid therapy, complete blood counts and hepatic/renal chemistries must be monitored serially to permit early detection and management of toxicity. When adverse reactions occur, the regimen should be modified according to severity—dose reduction, drug substitution, or institution of specific symptomatic measures—to preserve both safety and efficacy (160).

## 7 Future perspectives on the treatment of drug-resistant *Nocardia* infections

### 7.1 Unresolved clinical and scientific challenges

At present, the prevention, control, and treatment of drug-resistant *Nocardia* infections continue to encounter numerous bottlenecks that urgently require targeted research to fill the existing gaps in the field. At the clinical level, limited diagnostic capacity in primary-level hospitals leads to delayed therapy: current rapid technologies such as MALDI-TOF MS are restricted to large tertiary-center microbiology laboratories because of high instrument costs and the need for specialized maintenance, whereas conventional culture remains too slow-growing to provide timely results, causing primary-level patients to miss the optimal treatment window (97, 161).

No commercial *Nocardia* POCT kits exist, despite unmet needs for low-cost rapid screening in primary/secondary institutions (2). Emerging strategies focus on immunochromatographic assays targeting nocardomycolic acids and LAMP assays for 16S rRNA/*nbtS* genes (162), these approaches are designed for rapid turnaround (typically 45–70 min), low cost, and operation without specialized instrumentation, making them suitable for resource-limited settings.

For patients with end-stage renal disease (ESRD) on dialysis and pregnant individuals with nocardiosis, targeted clinical research is critical: (1) ESRD cohort: A multicenter, open-label trial (*n* = 80) comparing linezolid (600 mg once daily vs. 300 mg twice daily) for 8–12 weeks, with the primary endpoint of clinical cure rate and secondary endpoints including thrombocytopenia incidence (weekly platelet monitoring) and plasma trough concentrations (target 2–7 μg/mL) (163, 164); (2) Pregnant cohort: A prospective observational study (*n* = 30) of linezolid (600 mg every 12 h) vs. TMP-SMX (160/800 mg every 12 h + folate 5 mg daily) in the second and third trimesters, with the primary endpoint of maternal infection resolution and secondary endpoints of fetal adverse events (ultrasound at 20, 30, and 36 weeks) and infant developmental outcomes at 6 months (18). Among these populations, subsets with comorbidities or immunologic impairment experience significantly poorer outcomes, including severe complications or death, yet no robust data quantify how their treatment success rates differ from those of the general population (165, 166). At the research level, patients with *Nocardia* infection carry a high risk of co-infection with additional pathogens; in those with severe nocardiosis the rate reaches 44%. Common co-pathogens include filamentous fungi such as Aspergillus spp. and mycobacteria such as *Mycobacterium avium*. Yet these co-infections are documented mainly as single cases or small series, and the pathogenic mechanisms underlying polymicrobial involvement remain to be elucidated (156). However, reliable polymicrobial infection models are urgently needed to elucidate *Nocardia*-fungus/mycobacterium interactions: (1) Immunocompromised mouse model (C57BL/6, cyclophosphamide-induced neutropenia) co-infected with *N. farcinica* (1 × 10^6^ CFU intratracheal) and *Aspergillus fumigatus* (1 × 10^5^ conidia intratracheal), mimicking clinical pulmonary co-infection (167); (2) Outcome measures: survival rate, bacterial/fungal burden in lung tissue, cytokine profiling (TNF-*α*, IL-6, IL-1β) via ELISA, and histopathological analysis of granuloma formation (18); (3) Mechanistic studies: Transcriptomic analysis of *Nocardia* iron-acquisition genes (*nbtS*, *nbtB*) and *A. fumigatus* siderophore genes (*sidA*) to identify cross-species nutrient competition (168).

Published work has identified iron-acquisition genes as critical virulence determinants of *Nocardia*, and co-infection, especially with fungi, has been verified as an independent risk factor for poor prognosis in *Nocardia*-infected patients, whose clinical outcomes are significantly worse than those with monomicrobial infection; nevertheless, precise treatment-failure rates have yet to be quantified (156). Mechanisms underlying the dissemination of resistance genes remain insufficiently characterized. Although class 1 and class 3 integrons have been implicated in the horizontal transfer of these genes—being present in 93.42 and 56.57% of clinical isolates resistant to co-trimoxazole, respectively—Resistance gene transmission between environmental and clinical *Nocardia* requires targeted tracking studies: (1) Cross-regional surveillance across China, Australia, and Greece for *sul1*, *sul2*, and *dfrA(S1)* in environmental and clinical isolates (a single-center study detected *sul1* (9.37%), *sul2* (4.68%), *dfrA(S1)* (0%) in 79 isolates) (11); (2) MLST targeting *gyrB*/*secA1*/*hsp65* and WGS to identify shared resistance gene cassettes and integron-transposon structures (90, 169); (3) Mantel test to correlate genetic similarity with geographic proximity/antimicrobial use density and map transmission hotspots (170).

Novel antimicrobial targets against *Nocardia* are urgently needed to address the narrow therapeutic repertoire and rising resistance. Three preclinically validated targets stand out, each addressing a critical pathogenic pathway and offering translational potential: (1) Cell wall biosynthesis enzymes (KasA, MycP) are species-specific and essential for *Nocardia* survival, minimizing off-target toxicity (171); (2) Iron-acquisition proteins (NbtS, NbtB) are pivotal for immune evasion and persistent infection, with genetic deletion ablating *N. farcinica* neurovirulence *in vivo* (169); (3) ATP synthase subunit c is conserved across drug-resistant *Nocardia* strains, sharing high homology with mycobacterial AtpE (the bedaquiline target), supporting repurposing feasibility (172). The key translational challenge is target validation: high-throughput screening of compound libraries against these targets, combined with human-relevant infection models, is required to advance lead compounds to clinical trials. This virulence- and resistance-linked target prioritization addresses the root cause of treatment failure, rather than merely suppressing bacterial growth.

### 7.2 Research progress in the development of novel anti-*Nocardia* agents

The development of novel anti-*Nocardia* agents is pivotal to addressing drug resistance.

Bedaquiline (diarylquinoline inhibiting mycobacterial ATP synthase F1Fo) exhibits potent *in vitro* activity against NTM and is approved for multidrug-resistant *M. tuberculosis*. *In silico* analysis predicts binding to *Nocardia* F1Fo ATP synthase subunit c (≥70% homology with mycobacteria), but *in vitro* susceptibility testing of drug-resistant *Nocardia* isolates (TMP-SMX-resistant *N. farcinica*, imipenem-resistant *N. cyriacigeorgica*) is needed to validate its potential (173); however, no studies have yet evaluated its activity against *Nocardia* spp., and targeted experiments are therefore required to determine whether this compound exerts antibacterial efficacy against the genus.

In screens for agents with potential anti-*Nocardia* activity, several compounds have demonstrated promising antibacterial effects. Thiazolyl peptides such as nocathiacin and thiazomycin, together with their semi-synthetic analogues, exhibit potent activity against drug-resistant clinical isolates of *Mycobacterium tuberculosis*; although no data yet address their efficacy toward *Nocardia* spp., the pronounced inhibition they display against mycobacteria and other Gram-positive pathogens provides a rationale for exploring their development as novel anti-*Nocardia* agents (174, 175). In addition, natural-product screening has identified actinomycete-derived metabolites with broad-spectrum antibacterial activity. Strain *Nocardia* sp. UTMC 751, isolated from soil, produces five as-yet-uncharacterized metabolites that markedly inhibit *Staphylococcus aureus* and *Pseudomonas aeruginosa*. Given that *Nocardia* metabolites frequently exhibit potent antimicrobial properties, the secondary metabolites of this strain constitute a promising starting point for the development of novel antibacterial agents (176). These candidate scaffolds—bedaquiline (repurposing), thiazolyl peptides (optimization), and *Nocardia* sp. UTMC 751 metabolites (novel discovery)—represent complementary strategies to expand the therapeutic pipeline. However, their translation to clinical use requires addressing three interconnected barriers: (1) *In vitro*/*in vivo* efficacy against clinically relevant multidrug-resistant strains (e.g., sul1-positive *N. farcinica*, imipenem-resistant *N. cyriacigeorgica*) (169); (2) Pharmacokinetic optimization to enhance tissue penetration (e.g., blood–brain barrier for CNS infections) (177); (3) Safety profiling to mitigate adverse effects (e.g., ototoxicity of amikacin, myelosuppression of linezolid) (178). Prioritizing repurposing (e.g., bedaquiline) can accelerate clinical availability, while novel metabolite discovery offers long-term solutions for pan-resistant strains—creating a balanced pipeline that addresses both immediate and future clinical needs (179).

### 7.3 Innovative technologies for the treatment of drug-resistant *Nocardia*

Innovative technologies are demonstrating broad application prospects in the field of drug-resistant *Nocardia* therapy. At the diagnostic level, the continued development and refinement of NGS platforms are expected to markedly improve the accuracy and speed of *Nocardia* detection. For instance, whole-genome sequencing of *Nocardia* isolates enables precise species identification and comprehensive characterization of resistance and virulence determinants, thereby furnishing a solid foundation for precision therapy. Concurrently, mass spectrometry-based diagnostic approaches are being optimized: MALDI-TOF MS is increasingly used for *Nocardia* identification, and emerging mass spectrometric techniques hold promise for further enhancing both accuracy and efficiency.

On the therapeutic side, the integration of physical interventions with pharmacotherapy has opened new avenues for managing drug-resistant *Nocardia* infections. In cases of refractory *Nocardia* keratitis, for example, femtosecond laser-assisted lamellar keratectomy effectively removes infected tissue while simultaneously enhancing the corneal penetration of topical antibiotics, thereby markedly improving clinical outcome. Moreover, computer-aided drug-design strategies that exploit high-resolution structural data of *Nocardia*-specific proteins are yielding rationally tailored inhibitors, promising a next generation of highly effective agents. Collectively, these innovative technologies—enhanced NGS/MALDI-TOF MS, FLAK for refractory infections, and CADD—offer a transformative opportunity to address the diagnostic-therapeutic gap in drug-resistant nocardiosis. The critical insight is their synergistic integration: CADD identifies novel inhibitors targeting resistance/virulence pathways, while advanced diagnostics enable rapid speciation and resistance profiling to guide their use, and physical interventions overcome tissue penetration barriers. However, their widespread adoption is limited by cost (NGS/CADD) and technical expertise (FLAK), particularly in resource-limited settings. The forward-looking priority is to democratize these technologies—e.g., developing low-cost LAMP-based diagnostics, open-source CADD pipelines, and simplified FLAK protocols—ensuring that technological advances translate to equitable improvements in patient outcomes globally.

### 7.4 Prospects of multidisciplinary collaboration in the treatment of drug-resistant *Nocardia*

Optimal management of infections caused by drug-resistant *Nocardia* demands close multidisciplinary collaboration. Microbiological research underpins accurate species identification and elucidation of resistance mechanisms; genomic and proteomic analyses of resistance determinants and virulence-associated proteins provide specific targets for antimicrobial development. Medical imaging plays a pivotal role in both diagnosis and therapeutic monitoring: X-ray, computed tomography, and magnetic resonance imaging precisely delineate the site, extent, and severity of infection, supplying essential information for treatment planning and efficacy assessment.

Clinicians must integrate microbiological identification, imaging data, and individual patient characteristics to devise personalized therapeutic regimens. Clinical pharmacists, leveraging their expertise in antimicrobial selection, drug-interaction evaluation, and adverse-event monitoring, assist in refining these regimens to enhance safety and rationality. Furthermore, close collaboration between basic researchers and clinical teams accelerates the translation of bench discoveries to bedside application; Multidisciplinary collaboration should focus on a pivotal phase III RCT for novel agents: (1) Study design: Multicenter, double-blind, non-inferiority trial (n = 300) comparing novel thiazolyl peptide analogue (NTPA, 4 mg/kg qd iv) vs. linezolid (600 mg q12h iv) for 8 weeks in patients with severe pulmonary nocardiosis (immunocompromised or multidrug-resistant) (176); (2) Collaborating teams: Clinical microbiologists (species identification/susceptibility testing), infectious disease physicians (patient recruitment/monitoring), clinical pharmacists (TDM, target NTPA trough 8–12 μg/mL), and medical imaging specialists (CT-based lesion regression assessment) (180); (3) Endpoints: Primary: Clinical cure rate at week 12; secondary: relapse rate (6-month follow-up), adverse event rate, and microbiological eradication (negative sputum culture at week 8) (18). Multidisciplinary collaboration is not merely a supportive strategy but a necessity to overcome the complex, interconnected challenges of drug-resistant nocardiosis. The core value lies in integrating complementary expertise to close translational gaps: microbiologists define regional resistance profiles and validate diagnostic tools; infectious disease physicians design mechanism-based clinical trials and implement precision therapy; clinical pharmacists optimize dosing and monitor adverse effects; basic researchers identify novel targets and develop human-relevant models; and medical imaging specialists provide non-invasive response monitoring. A paradigmatic example is the phase III RCT for novel thiazolyl peptide analogues—this trial cannot succeed without microbiological support for susceptibility testing, pharmacokinetic guidance for dosing, and imaging for efficacy assessment. By aligning these efforts around prioritized research gaps (e.g., POCT development, special population trials), multidisciplinary teams can transform descriptive knowledge into actionable clinical solutions, accelerating the translation of bench discoveries to bedside impact and reducing the global burden of drug-resistant nocardiosis.

## 8 Prevention strategies for drug-resistant *Nocardia* infections

Given the rising incidence of drug-resistant *Nocardia* infections and limitations in therapeutic options, targeted prevention strategies are critical to reducing disease burden. These strategies should integrate infection control, environmental exposure mitigation, targeted screening, and antimicrobial stewardship, tailored to high-risk populations and regional epidemiological profiles.

### 8.1 Infection control measures in healthcare settings

Healthcare-associated *Nocardia* infections (e.g., post-surgical, catheter-related) account for 10–20% of cases PMC, primarily linked to contaminated medical devices or environmental surfaces (42). Key control measures include (GRADE: Moderate): (1) Rigorous disinfection of respiratory equipment (e.g., ventilators, nebulizers) with hydrogen peroxide vapor or peracetic acid, as *Nocardia* can persist on dry surfaces for up to 7 days (42); (2) Surveillance of healthcare workers with chronic pulmonary diseases, who are at increased risk of colonization and transmission (181); (3) Isolation of immunocompromised patients with active nocardiosis to prevent cross-infection, as aerosolized droplets may facilitate transmission PubMed (18).

### 8.2 Mitigation of occupational and environmental exposure

*Nocardia* is ubiquitously present in soil and water, making occupational groups (farmers, gardeners, construction workers) and individuals with frequent outdoor exposure at higher risk (18, 182). Prevention measures(GRADE: Low) include: (1) Use of personal protective equipment (PPE) such as gloves, masks, and eye protection during soil handling or construction activities; (2) Avoidance of direct contact with contaminated water sources (e.g., stagnant ponds) in endemic regions CDC; (3) Improved ventilation in agricultural or industrial settings to reduce inhalation of aerosolized spores. For immunocompromised individuals, environmental modifications include avoiding indoor potted plants and humidifiers, which can serve as reservoirs for *Nocardia* (183).

### 8.3 Targeted screening in high-risk populations

Early detection of colonization or subclinical infection in high-risk groups can prevent progression to severe disease. Recommended screening strategies(GRADE: Moderate) (3, 18, 184):(1) Annual sputum culture and 16S rRNA gene PCR for cystic fibrosis (CF) patients, among whom *Nocardia* colonization prevalence reaches 4–12%; (2) Pre-transplant screening of solid organ and hematopoietic stem cell transplant (SOT/HCT) recipients, with follow-up screening at 3, 6, and 12 months post-transplant; (3) Periodic chest CT and microbiological testing for patients on long-term immunosuppressive therapy (e.g., rheumatoid arthritis, systemic lupus erythematosus). Positive screening results should prompt close clinical monitoring rather than empirical therapy, as colonization does not always indicate active infection.

### 8.4 Policy-driven antimicrobial stewardship

Antimicrobial overuse is a key driver of *Nocardia* drug resistance (18). Stewardship strategies (GRADE: High) include: (1) Regional guidelines tailored to local resistance (e.g., restricting third-generation cephalosporins in sub-Saharan Africa (*β*-lactam resistance 35–42%) (47); limiting TMP-SMX prophylaxis in Australian (8.3% transplant-associated resistance) and Chinese (11.7% resistance, 91.2% with *sul1*/*sul2*) transplant patients) (6); (2) Mandatory AST for all *Nocardia* isolates (89% of labs follow CLSI standards) (185); (3) Clinician education on rational combination therapy (reduces resistance by 47% vs. monotherapy) (186). WHO GLASS should incorporate *Nocardia* into routine surveillance (187).

## 9 Conclusion

Drug-resistant *Nocardia* infections represent a growing global clinical challenge, characterized by marked regional heterogeneity in species distribution and antimicrobial susceptibility. This review consolidates the latest evidence, confirming *N. farcinica* as the dominant species in China, the *N. nova* complex in Australia, and *N. brasiliensis* in tropical regions—patterns that directly inform empirical therapy.

Diagnostically, culture remains the gold standard but is hampered by slow turnaround, while MALDI-TOF MS and NGS offer rapid species and resistance detection but are limited by resource barriers. Therapeutically, linezolid demonstrates universal *in vitro* activity and is preferred for severe infections, whereas TMP-SMX and amikacin remain core agents, requiring individualized dosing and therapeutic drug monitoring in special populations. Prevention strategies, including infection control and antimicrobial stewardship, are supported by preliminary evidence but require regional adaptation.

Future directions: (1) Develop low-cost POCT assays targeting *Nocardia*-specific genes; (2) Conduct multicenter trials to validate combination therapy for multidrug-resistant strains; (3) Advance novel therapeutic targets (e.g., NbtS, KasA) and drug repurposing (e.g., bedaquiline); (4) Integrate *Nocardia* into global antimicrobial resistance surveillance systems (e.g., WHO GLASS).

Recommendations: (1) Mandate susceptibility testing for all isolates to guide empirical therapy; (2) Implement individualized regimens for high-risk patients; (3) Enforce rigorous infection control in healthcare settings; (4) Restrict overuse of broad-spectrum antibiotics via regional stewardship; (5) Foster multidisciplinary collaboration to bridge bench-to-bedside gaps.

In conclusion, optimizing the management of drug-resistant *Nocardia* infections requires synergistic, regionally tailored strategies integrating epidemiology, rapid diagnostics, evidence-based therapy, and targeted prevention. Addressing these priorities will be pivotal to reducing global disease burden and improving patient outcomes.
